# The association between IUGR and maternal inherited thrombophilias: A case-control study

**DOI:** 10.1097/MD.0000000000012799

**Published:** 2018-10-12

**Authors:** Stefan Dugalić, Milos Petronijevic, Aleksandar Stefanovic, Katarina Jeremic, Svetlana Vrzic Petronijevic, Ivan Soldatovic, Igor Pantic, Irena Djunic, Zoran Jokic, Filip Djokovic, Jelena Dotlic, Milica Zaric, Jovana Todorovic

**Affiliations:** a Clinical Center of Serbia, Clinic for Gynecology and Obstetrics; b School of Medicine, University of Belgrade; c Institute of Medical Statistics and Informatics; d Institute of Medical Physiology Rihard Burijan; e Clinical Center of Serbia, Clinic for Hematology, Belgrade; f Faculty of Health, Legal and Business Studies, Singidunum University, Valjevo; g Institute of Epidemiology; h Institute of Social Medicine, Belgrade, Serbia.

**Keywords:** inherited thrombophilia, obstetric vascular complications, risk factors, unknown cause IUGR

## Abstract

One of the risk factors for vascular obstetric complications, such as intrauterine growth restriction (IUGR), is inherited thrombophilias. Nevertheless, routine screening for thrombophilias is not endorsed in pregnant women due to their low prevalence and conflicting results of published studies regarding the usefulness of screening in these patients. The cause of IUGR remains unknown in almost 1 quarter of cases. There are no published studies evaluating the association of inherited thrombophilias and IUGR in patients with IUGR of unknown origin. Understanding and preventing IUGR is an important public health concern, as IUGR has been associated with fetal mortality and neonatal morbidity, as well as adverse long-standing consequences. This study aimed to evaluate the prevalence of inherited thrombophilias in IUGR of unknown cause and to test the association between the inherited thrombophilias and IUGR of unknown cause.

This study included 33 cases of IUGR of unknown cause tested for inherited thrombophilias and 66 controls individually matched for age, ethnicity, and smoking status.

Patients with plasminogen activator inhibitor 1 (PAI-1) and methylenetetrahydrofolate reductase (MTHFR) had significantly higher odds for IUGR of unknown cause (*P* < .001 and *P* = .002, respectively) with OR 13.546 (CI 95% 3.79–48.37) and 8.139 (CI 95% 2.20–30.10), respectively. A positive association between other inherited thrombophilias (homozygous 20210 prothrombin gene mutation and homozygous factor V Leiden) and IUGR of unknown cause was also found, *P* = .096, OR 6.106 (CI 95% 0.72–51.30), although it was not statistically significant (*P* = .096, OR = 6.106, CI 95% 0.72–51.30).

Our results indicate that PAI-1 and MTHFR thrombophilias represent risk factors for IUGR of otherwise unidentified cause.

## 1 Introduction

The establishment and adequate maintenance of placental circulation is *conditio sine qua non* for a successful and uneventful pregnancy. Thrombophilia appears as a risk factor of placental vascular disorders (comprising both the decidua and the spiral arteries) and the presence of secondary thrombosis with hypercoagulability which could lead to impairment of maternal-fetal circulation.^[1]^ Therefore, thrombophilias could be responsible for some important obstetric vascular complications, such as intrauterine growth restriction (IUGR), preeclampsia (PE), placental abruption, recurrent miscarriages, intrauterine fetal death, and unexplained stillbirth which is supported by growing body of evidence.^[2,3]^ And indeed, thrombophilia has been confirmed in 65% of pregnant women with these complications.^[4]^ Systematic reviews and meta-analyses indicate that women with thrombophilia are at increased risk of IUGR.^[5,6]^ The underlying placental pathology in thrombophilia resembles that seen in other pregnancy disorders related to chronic obstruction of the maternal or fetal vasculature. Although particular placental lesion is no pathognomonic for thrombophilia, lesions that could reveal maternal thrombotic disease include decreased placental weight (placentas with weight small for gestational age (<10th percentile)), infarcts, increased numbers of syncytial knots, “accelerated villous maturation” and atherosis.^[7–9]^ For all these reasons, testing for inherited thrombophilias in cases of severe IUGR is recommended by some professional organizations, such as The American College of Obstetrics and Gynecology (ACOG).^[10]^

However, in cases of nonsevere IUGR, situation is unclear. Routine screening for thrombophilias in women with IUGR is not endorsed due to low prevalence of thrombophilias and because of the conflicting results of performed studies.^[11]^ Large case-control performed in 493 cases and 472 controls found no increased risk of IUGR in women with thrombophilias, excluding for a subcategory of women with the methylenetetrahydrofolate reductase (MTHFR) variant.^[12]^ A meta-analysis of case-control studies performed by Howley et al and Facco et al found a significant relationship between factor V Leiden (FVL), the prothrombin gene variant (PT), MTHFR, and IUGR.^[5,13]^ Still, even the authors suggested that perceived strong association could be driven by small, poor-quality studies that yield extreme associations,^[5]^ or the presence of publication bias was suggested by funnel plot analysis due to the small number of negative studies.^[13]^

Although fetal (chromosome disorders, congenital anomalies, infections), maternal (genetic factors, nutritional status, chronic diseases resulting in uterine ischemia, or hypoxia) and placental (chorioangioma, villitis, ischemic villous necrosis) clinical circumstances disturb fetal growth and explain most of the occurrences of IUGR, just about 25% of causative factors remain unknown.^[14]^ Studies on fetal programing have clearly demonstrated the presence of fetal growth epigenetics linked with adverse long-standing consequences.^[15,16]^ Therefore, determination of the reasons for IUGR in those 25% of cases with unidentified causes becomes an important goal for the obstetricians, since the understanding and preventing IUGR is of public health importance. To the best of our knowledge, association between thrombophilias and IUGR in asymptomatic women with IUGR has not been tested so far. All of the previous studies have evaluated relationship between thrombophilias and all newborns with IUGR, but not have examined association between exclusively IUGR of unknown etiology and thrombophilias. Although MTHFR has been found to be the most prevalent thrombophilias in pregnancy,^[11,17]^ previous studies have focused mainly on factor V Leiden/prothrombin G20210A carriers since these are thought to be the most thrombogenic inherited thrombophilias.^[18]^

Since the IUGR with unrevealed cause is found frequently, we aimed to compare the prevalence of thrombophilias (especially MTHFR related) in IUGR cases with unknown cause and in uneventful pregnancies and to test the association between trombophilias and IUGR of unidentified cause. As IUGR and inherited thrombophilias are both rare, we decided to perform case control study.

## 2 Methods

This case-control study analyzed the prevalence of inherited thrombophilias among women with IUGR of unknown cause (cases) and among women with uneventful pregnancies (controls) and tested association of inherited thrombophilias with IUGR of unknown cause among women delivered in Clinic for Gynecology and Obstetrics, Clinical Center of Serbia (the biggest clinic in Serbia, with roughly 7000 deliveries per year), from December 2014 to December 2017. IUGR is one of the most common reasons for referral to our Clinic. Identified fetuses with < 10th percentile weight for gestational age are monitored for fetal growth and fetal physiology over time in our Clinic. A normal growth trajectory, normal Doppler velocimetry of the umbilical artery and normal amniotic fluid volume suggests a constitutionally small fetus or minimal fetal impact from uteroplacental insufficiency and those patients are discharged from clinic and followed as outpatients and those were not included in study. In cases of confirmed IUGR, we applied protocols similar or identical to Stage-Based Management Protocol proposed by Gratacós and Figueras.^[19]^

Eligibility criteria were singleton live birth with prenatally diagnosed IUGR of unknown cause (confirmed by the birth of newborns with birth weight below the 10th percentile for gestational age and sex) and performed testing for inherited thrombophilias: Prothrombin G20210A mutation, Factor V Leiden mutation, Homozygosity to MTHFR C677T, Homozygosity to 4G/4G mutation in plasminogen activator inhibitor 1 (PAI-1) gene mutation. Exclusion criteria were maternal systemic conditions (pregestational diabetes class C, D, R, and F, chronic hypertension, chronic renal diseases, systemic lupus erythematosus, antiphospholipid syndrome), chronic maternal hypoxemia (due to cardiac disease, pulmonary disease, or hematologic disorders), maternal malnutrition and gastrointestinal conditions/diseases, short interpregnancy interval, uterine factors (large fibroids, Mullerian anomalies), substance abuse problem (Maternal illicit drug use, excessive consumption of alcohol), and pregnancies achieved by assisted reproduction techniques. Controls were women with uneventful singleton pregnancies who gave birth at our Clinic and met the same eligibility and exclusion criteria, except for IUGR.

Since the only inherited thrombophilias that are not affected by pregnancy and that can be adequately tested for in pregnancy are FVL and PT gene mutations,^[10]^ all case subjects were selected amongst those women with IUGR in index pregnancy who were tested for inherited thrombophilias at least 8 weeks postpartum. Consequently, all controls were chosen amongst those women tested for inherited thrombophilias before the index pregnancy according to the antenatal screening guidelines (personal or family history of venous thromboembolism, increased age, obesity, smoking, and obstetric risk factors such as recurrent pregnancy loss, previous pregnancies with placental abruption, preterm birth, or pre-eclampsia).^[20]^ All case subjects were tested for inherited thrombophilias in the puerperal period in DiaLab Laboratory, and included testing for FVL, PAI-1, MTHFR, and PT gene mutation. Testing was performed on genetic analyzer ABI 3130—Applied Biosystems; kit Elucigene TRP-F Thrombosis Risk Panel + PAI-1.

Case group consisted of 33 subjects with IUGR of unknown cause and control group encompassed 66 women with uneventful pregnancies. To avoid selection bias we have applied necessary principles in design of case control studies.^[21,22]^ Therefore, in the selection of cases we have clearly defined what case is (women with unexplained IUGR who has performed testing for inherited thrombophilias), applied objective criteria for the diagnosis of the condition under the study (IUGR),^[19]^ and included all incident study cases who developed the condition (IUGR) during the study period in our Clinic. Furthermore, we have applied the same eligibility criteria to the controls. Moreover, selection of controls is representative of the population that produced the cases (the same geographical area where the study subjects live, the same race, the same clinic, etc.). The rational for choosing controls among women who had been already prenatally tested for thrombophilia is based on the fact that the chances for inherited thrombophilias are lower in low risk women and doing test for thrombophilia polymorphism is expensive. In efforts to address other potential sources of bias, we performed individual matching. To achieve that controls are similar to the cases, we implemented individual matching for time of hospitalization (from December 2014 to 2017), age, ethnicity, and smoking status. Due to concern for sufficient numbers in stratified analysis and to add power to study given the expected low prevalence of inherited thrombophilias among controls, we have chosen higher matching schemes.^[23]^ Each case was matched with 2 healthy women who had at least 1 normal pregnancy and who delivered within the study period and those women formed control group (66 women).

A sample size of 99 (33 and 66) participants would result in over 80% power to detect an odds ratio of 1.5 at the 5% level of significance. Two groups were compared using Student *t* test, Fisher exact test, and Pearson *χ*^2^ test. Exact *P* values were used where appropriate. Relationship between binary dependent variable and independent variables is examined using logistic regression analysis. All data were analyzed using SPSS 20 (IBM Corp). All *P* values less than .05 were considered significant.

Statistical analyses were performed with the SPSS software for Windows version 6 (SPSS, Chicago, IL). Values were considered significant at *P* < .05.

This study was approved by the Professional Meeting of Clinic for Gynecology and Obstetrics, Clinical Center of Serbia (decision number 3802/12/13/17) and by the Ethical Committee Medical Faculty University Belgrade (decision number 29/XII/18).

The Institutional review board permitted the study without the patients’ informed consent because the study was based on retrospective design. Furthermore, study evaluated existing data recorded before the start of the study, all data was gathered with the respect of patients’ privacies and anonymity. Database does not contain any distinguishable information.

## 3 Results

**Figure 1:**
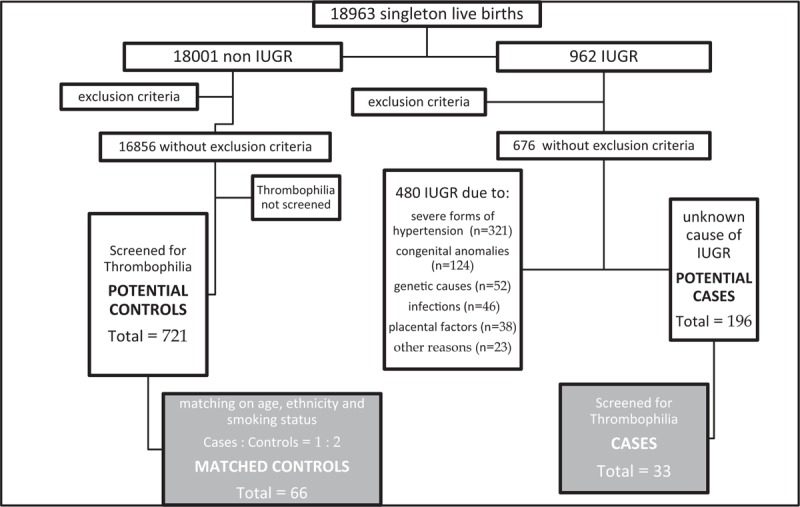
Flow chart diagram.

During the study period, among 18,963 deliveries of women with singleton pregnancies, 5.07% were with IUGR. After implementation of exclusion criteria, we found 676 IUGR subjects. Among them in 71.01% subjects, the cause of IUGR was determined, while 28.99% were with unidentified cause of IUGR. The identified causes of IUGR and other data regarding the selection of cases and controls are presented in flow chart diagram (Fig. 1). After implementing both exclusion and eligibility criteria we found 33 cases and 721 potential subjects for control group and among them 66 were selected as matched controls (Fig. 1).

Significant differences were not find regarding the characteristics of subjects in case and control group, except those expected to be different due to eligibility criteria used for the formation of both groups and their consequences (gestation week at delivery, mode of delivery, birthweight, and Apgar score). This data are presented in Table 1.

**Table 1:**
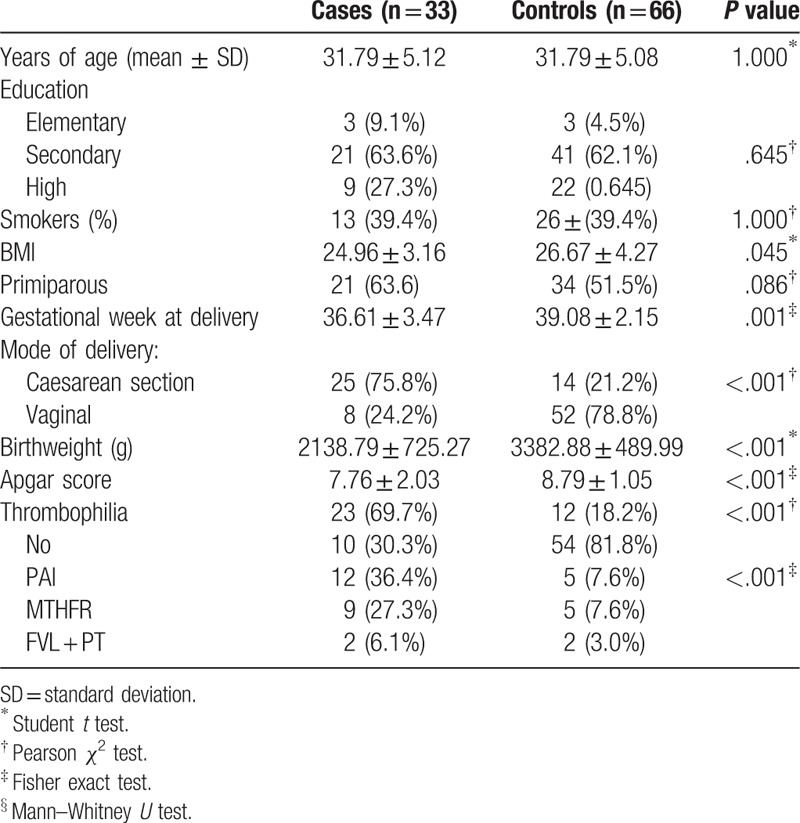
Characteristics of the study participants.

In our case-control study 23 (69.69%) patients with IUGR of unknown cause (case group) and 12 (18.88%) with normal fetal growth had inherited thrombophilias (Table 2). We found a statistically significant association between IUGR of unknown cause and PAI-1 gene mutation and MTHFR. Although not statistically significant, positive association between other inherited thrombophilias (homozygous 20210 prothrombin gene mutation + homozygous FVL) and IUGR of unknown cause was also found. However, the probably reason for failure to reach statistically significant association lies in the fact that in our study we had few participants with FVL and PT gene mutations. These data and frequencies of different types of inherited thrombophilias are presented in Table 2.

**Table 2:**
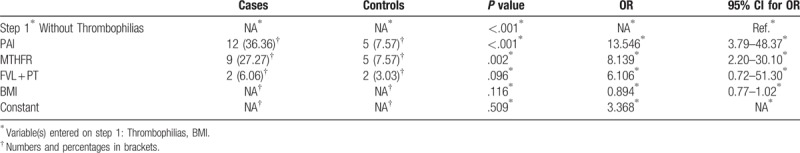
Variables in the equation.

This analysis was made with adjustment for BMI. The stated variability of the Nagelkerke R square is 0.351 and the classification power of the model is 77.8%.

**Figure 2:**
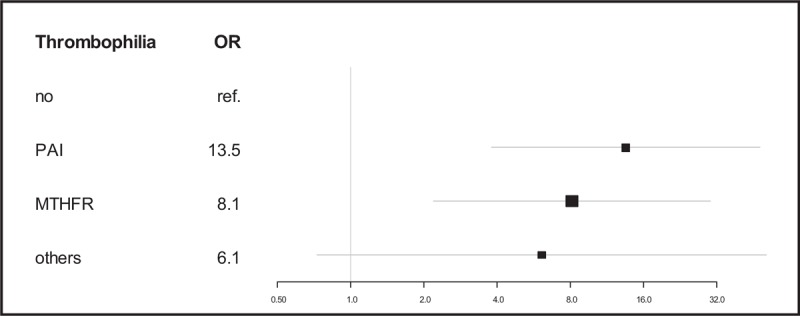
Inherited thrombophilias as risk factors associated with IUGR of unknown cause. IUGR = intrauterine growth restriction.

Strongly significantly higher odds for IUGR of unknown cause are present in patients with PAI and MTHFR thrombophilias (Fig. 2).

## 4 Discussion

This case-controlled study demonstrated significantly higher prevalence of inherited thrombophilias in women with IUGR of unknown cause compared with women with uneventful pregnancies. Furthermore, strongly significantly associations between PAI and MTHFR thrombophilias and IUGR of unknown cause are demonstrated.

We found higher prevalence of inherited thrombophilias in case group compared with the controls, which is in accordance with results of meta-analysis performed by Alfirevic et al^[24]^ and other case-controlled studies.^[25]^ Still, the prevalence of all inherited thrombophilias in study of Jamal et al was 55.9% in the case group compared with 10.3% in the control group, which is much lower than prevalence in our study groups. Dissimilarities between these results could arise from variances among our study participants and those evaluated by Jamal et al. Their cases encompassed all IUGR subjects, while our included only those with unknown cause of IUGR. In addition, their controls were allocated from general population of women, where the prevalence of inherited thrombophilias is higher compared with population of high risk women for thrombophilias from where our controls were recruited. Furthermore, we are aware of the fact that the prevalence of all thrombophilias and different kinds of inherited thrombophilias are dependent on race and ethnic origins. In East Asian populations, Protein C and Protein S deficiencies are much more prevalent than MTHFR, prothrombin, and FVL mutations,^[26,27]^ which is not the case in Europe.^[24]^

A significant relationship between IUGR of unknown cause and PAI-1 and MTHFR gene mutation found in our study is in line with the results of the majority of other case-controlled studies which evaluated association of these conditions in general population of women with IUGR.^[28,29]^ On the contrary, Said et al found no link between IUGR and PAI-1 and MTHFR gene mutations.^[30]^ However, these discrepancies could be explained by huge differences regarding race and ethnicity between participants in conflicting studies.

To the best of our knowledge, our study is the first one to appraise the association between inherited thrombophilias and IUGR of unknown cause. Furthermore, numerous and very strict exclusion criteria enabled the selection of homogenous groups of study participants, thus eliminating a large number of potential confounders. In addition, we took care for the way of appointing the cases with the intention of avoiding biases. We used incident rather than prevalent cases for depicting the study subjects. Case subjects were women without risk factors for inherited thrombophilias prior to index pregnancy complicated with IUGR. Furthermore, in selecting control subjects, exposure to risk factors, and confounders were typical of that in the population “at risk” of becoming cases—to be precise, people who do not have the evaluated condition under investigation, but who would be encompassed in the study as cases if they had. Besides, we performed individual matching to additionally eliminate confounding and even performed additional statistical adjustment for confounders. Additionally, our study estimated the relation between inherited thrombophilias and IUGR of unknown cause exclusively by reviewing the medical histories, records and notes of cases and controls subjects managed in a single center. Since our center is the biggest and the most prominent tertiary health care center in Serbia, medical records record keeping is uniform, adequate or reliable, providing that evaluated data are more accurate than those data that depends on memory of patients or inconsistent data obtained from multiple health care centers.

However, the number of studied cases was limited by the rarity of the conditions that were under investigation and we acknowledge this as limitation of the study. Besides, controls were depicted from general population of women, but from the high-risk population for inherited thrombophilias since the current guidelines for antenatal screening for inherited thrombophilias in low risk population are not recommended and such screening is expensive.^[20]^ This is additional limitation of this study.

In conclusion, we might say that some types of inherited thrombophilias, such as PAI and MTHFR, present risk factor for IUGR of unknown cause. Larger, prospective studies are needed to have additional insight into the association of these to conditions and to perform randomized controlled trials to evaluate the potential benefits of administration of low-dose aspirin and/or heparin in cases of IUGR of unknown cause in women with inherited thrombophilias.

## Acknowledgment

The authors are grateful for the assistance of Dia Lab Policlinic for Laboratory Diagnostics, Radoslava Grujića 1, Belgrade, Serbia in the performance of testing for FVL, PAI-1, MTHFR, and PT gene mutation in case group of patients.

## Author contributions

SD: study design, data collection, data interpretation, writing and revising the manuscript; MP: data collection, data interpretation, revising the manuscript; AS: data collection, data interpretation, writing the manuscript; KJ: data collection, data interpretation, writing the manuscript; SVP: data collection, data interpretation, writing the manuscript; BM: study design, data collection, data interpretation; IS: data interpretation, statistical analysis, writing the manuscript; ID: study design, data interpretation, statistical analysis, writing the manuscript; ZJ: study design, data interpretation, statistical analysis, writing the manuscript, revising the manuscript; DjP: study design, data interpretation, statistical analysis, writing the manuscript, revising the manuscript, supervision of the research group. All authors read and approved the final manuscript.

**Conceptualization:** Milos Petronijevic, Jovana Todorovic.

**Data curation:** Milos Petronijevic, Aleksandar Stefanovic, Katarina Jeremic, Svetlana Vrzic Petronijevic, Igor Pantic, Jelena Dotlic, Milica Zaric.

**Formal analysis:** Ivan Soldatovic, Milica Zaric.

**Investigation:** Stefan Dugalic, Aleksandar Stefanovic.

**Methodology:** Stefan Dugalic, Ivan Soldatovic, Igor Pantic, Zoran Jokic, Filip Djokovic, Jelena Dotlic, Jovana Todorovic.

**Resources:** Stefan Dugalic, Aleksandar Stefanovic, Igor Pantic, Irena Djunic, Zoran Jokic, Filip Djokovic, Jelena Dotlic, Milica Zaric.

**Software:** Ivan Soldatovic, Jelena Dotlic.

**Supervision:** Milos Petronijevic.

**Validation:** Milos Petronijevic, Katarina Jeremic, Svetlana Vrzic Petronijevic, Jovana Todorovic.

**Visualization:** Stefan Dugalic.

**Writing – original draft:** Stefan Dugalic.

**Writing – review & editing:** Stefan Dugalic, Milos Petronijevic, Aleksandar Stefanovic, Igor Pantic.
